# Immunotherapy role in bladder cancer treatment: a review of literature

**DOI:** 10.3389/fonc.2026.1908307

**Published:** 2026-09-02

**Authors:** Nishanth Thalambedu, Krishna Vasipalli, Nikhila Sampath Kumar, Sruthi Vellanki, Anup Kumar Trikannad, Manoja Gullapalli, Shi Ming Tu, Anuradha Kunthur

**Affiliations:** 1 Department of Hematology and Oncology, University of Arkansas for Medical Sciences, Little Rock, AR, United States; 2 Department of Internal Medicine, The University of Alabama at Birmingham, Birmingham, AL, United States; 3 Department of Hematology and Oncology, Renown Medical Group, Reno, NV, United States

**Keywords:** antibody drug conjugate (ADC), artificial intelligence, bladder cancer, CtDNA, immunotherapies

## Abstract

Immunotherapy has changed the landscape of bladder cancer treatment in recent years, both for localized and metastatic bladder cancer. While bacillus Calmette–Guérin (BCG) remains the gold standard for high-risk non–muscle-invasive bladder cancer, the development of checkpoint inhibitors continues to increase treatment options for patients who are intolerant or unresponsive to BCG. In the setting of muscle-invasive bladder cancer, perioperative checkpoint blockade has shown promising pathologic response rates and survival signals, especially in PDL1/CPS enriched populations. In metastatic bladder cancer, anti–PD-1/PD-L1 therapy can lead to durable responses in a subset of patients, and combination strategies, sequences of therapy, and biomarkers are all being explored to help guide patient selection and improve clinical outcomes. Herein, we review evidence supporting the use of immunotherapy in non-muscle invasive, muscle invasive, and metastatic bladder cancer including the newer immunotherapy combination approaches.

## 1 Introduction

Bladder cancer is one of the most common malignancies worldwide, resulting in remarkable morbidity and mortality, particularly in advanced stages where there are few therapeutic options (1, 2). In the United States alone, there are approximately 80,000 new cases and 18,000 deaths annually from bladder cancer. (Jalal et al., 2026) Most cases are urothelial carcinoma with a high tumor mutational burden (TMB) resulting in a higher neoantigen load and increased immunogenicity, making bladder cancer patients ideal candidates for immunotherapy (3, 4).

In bladder cancer, the tumor immune microenvironment (TME) is a complex multicellular ecosystem that not only consists of tumor cells, but also immune effector and suppressor cells, stromal cells, cytokines and extracellular matrix molecules that have a profound influence on tumor growth, progression, and therapeutic response (5). Tumor-infiltrating lymphocytes are crucial for mounting effective anti-tumor responses; however, their activity is often blocked by immunosuppressive molecules within the bladder TME (6–8). A high density of Tregs and M2 TAMs in the outer layers of the tumor parenchyma is insufficient for tumor rejection and is linked to poor survival rates (9).

From a molecular perspective, immune checkpoint pathways such as the programmed cell death-1 (PD-1)/programmed cell death-ligand 1 (PD-L1) axis are important regulators that limit anti-tumor T cell proliferation and activation in bladder cancer by triggering inhibitory signaling cascades. Blockade of the PD-1/PD-L1 pathway has become the basis of immunotherapeutic strategies for advanced bladder cancer. Novel molecular targets beyond the PD-1/PD-L1 checkpoint, including LAG-3, TIM-3, CTLA-4, and new innate checkpoints are being investigated (10).

Manipulation of the immune microenvironment is a major focus of ongoing bladder cancer research, and it is likely that the importance of immune-based therapy will only increase in the future. In this review, we will describe our current understanding of the pathophysiology of the immune system in bladder cancer and summarize the clinical data supporting use of established and novel immunotherapies, including BCG, immune checkpoint inhibition, vaccines, and other immune modulatory agents.

## 2 Immunological pathophysiology of bladder cancer

### 2.1 Immune checkpoints and cytokine signaling

The immune-checkpoint pathway that has been most studied in bladder cancer is PD-1/PD-L1, which is also the target of five currently approved immune checkpoint inhibitors (10). Binding of PD-1 to its ligands results in inhibition of T cell proliferation and reduction of tumor cell killing (11). Cancer- associated fibroblast (CAF) in the TGF-beta signaling pathway is the central mechanism that shapes the tumor microenvironment. TGF-beta signaling activated CAF results in tumor progression (12). IL-6 and IL-8 are also immunosuppressive through STAT3 signaling, while IFN-λ type III interferons are associated with increased phagocytosis by macrophages, high lymphocyte infiltration of tumors, and predict better responses to immune checkpoint inhibitors (13). Adenosine pathway activation by hypoxia and metabolic stress in the TME are important for immunosuppression via activation of A2a adenosine receptors on T cells, and A2a inhibitors are currently being tested for immunotherapy (14). Dysregulated myeloid-derived inflammatory cytokine productions and changes of macrophage polarization in the TME are frequently observed in bladder tumors. Macrophage secreted cytokines can upregulate PD-L1 expression on the tumor cells. M2-polarized TAMs release immunosuppressive mediators, further enhancing immune suppression (15).

### 2.2 Metabolic barriers and stromal components

Hypoxia in the TME impairs T cell functions through the hypoxia-inducible factor (HIF) pathway which upregulates immune checkpoints as well as CD39, CD73 and adenosine pathway activation (16). The acidic medium in the TME significantly affects immune checkpoint expressions in CD8+ T cells by increasing TIM-3 and LAG-3 and decreasing CTLA-4. Collagen fibers and hyaluronic acids serve as structural barriers in the TME against the immune cells. FGFR3 alterations in bladder cancer upregulates serine synthesis, which impairs macrophage activity and leads to decreased IFNⲁ/β production. Cancer-associated fibroblasts (CAFs) also have immunosuppressive functions by JNK activation, and JNK inhibition increases CD8+ T-cell number and restores function to enhance anti-PD-1 activity (12). CAFs recruit immunosuppressive lipid-associated macrophages via CXCL12 secretion. Uptake of environmental nutrients required for energy metabolism by exhausted T cells, and competition for nutrients by cancer cells and myeloid cells, are critical barriers for optimal anti-tumor immunity for cytotoxic lymphocytes (17). Dendritic cells (DC), bridges between the innate and adaptive immune systems, play a critical role in activating the anti tumor immunity but frequently compromised by the immunosuppressive microenvironment (9).

### 2.3 Microenvironment heterogeneity and clinical implications

**Figure 1:**
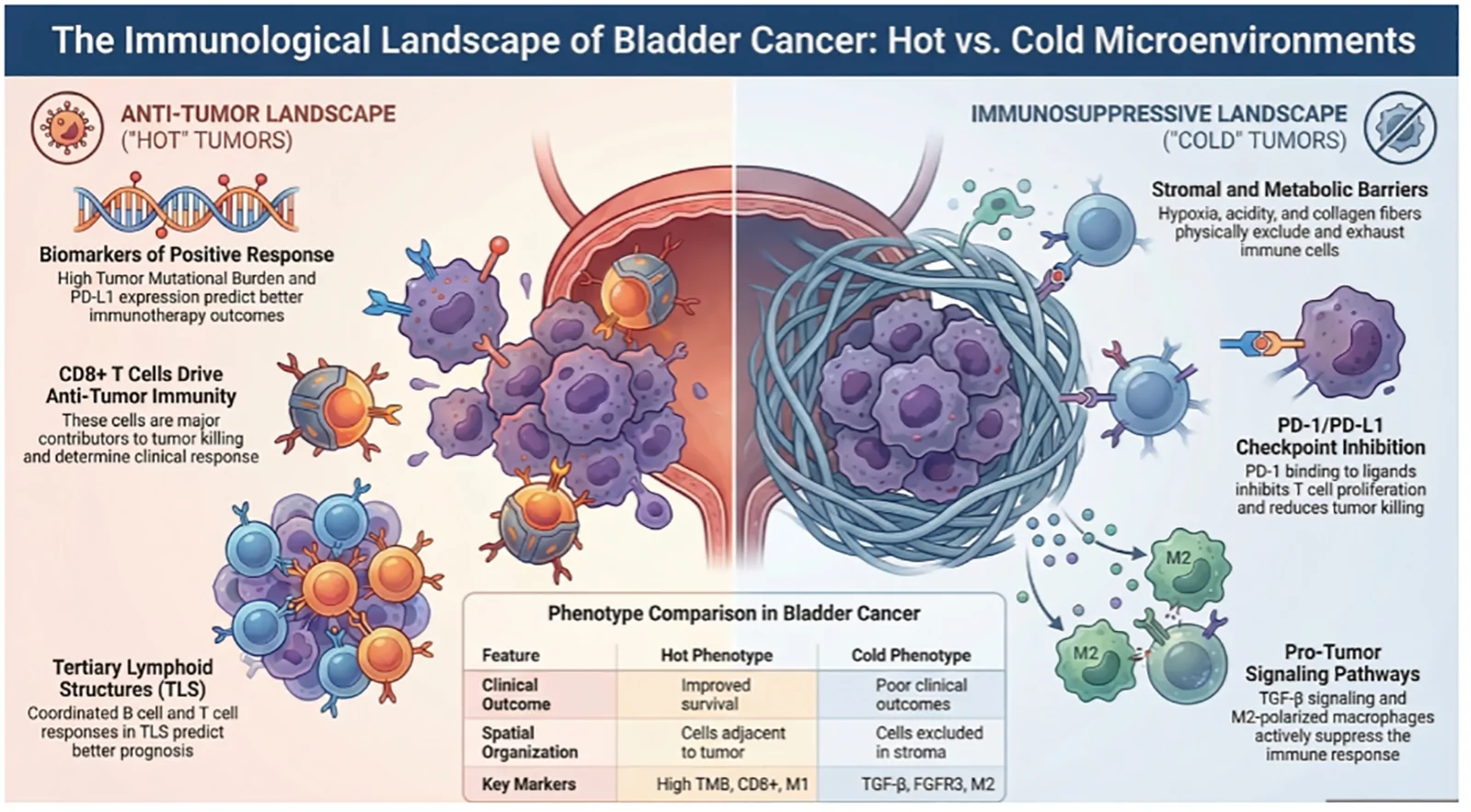
Mechanisms of action within the bladder cancer tumor immune microenvironment (TME). This schematic illustrates the contrasting immune phenotypes observed in bladder cancer. Hot tumors (left) are characterized by abundant infiltration of CD8^+^ cytotoxic T cells, tertiary lymphoid structures (TLS), and high expression of immune activation biomarkers, including PD-L1, which are associated with enhanced anti-tumor immune responses and improved responsiveness to immune checkpoint inhibitors. In contrast, cold tumors (right) exhibit immune exclusion, dense stromal and extracellular matrix barriers, hypoxia, and an immunosuppressive milieu enriched with M2-polarized tumor-associated macrophages (TAMs) and TGF-β signaling, resulting in impaired T-cell infiltration and reduced response to immunotherapy. The central table summarizes the major clinicopathologic and molecular differences between hot and cold bladder cancer phenotypes. BC, bladder cancer; TME, tumor microenvironment; CD8, cluster of differentiation 8; TLS, tertiary lymphoid structures; PD-1, programmed cell death protein 1; PD-L1, programmed death-ligand 1; TGF-β, transforming growth factor-beta; TAMs, tumor-associated macrophages; FGFR3, fibroblast growth factor receptor 3; TMB, tumor mutational burden; IFN-γ, interferon-gamma.

The immune phenotypes of bladder cancer span from hot and inflamed with high infiltration of activated immune cells (which has better clinical outcomes) to cold and non-inflamed (which has worse clinical outcomes). This dichotomy of clinical outcomes between hot and cold immune phenotypes can be translated to better clinical responses to immune checkpoint inhibitor therapy by tumors with hot TME (18). In contrast, tumors with cold TME and enriched for high TGF-β signaling and FGFR3 mutations are resistant to ICI treatments (19). Spatial organization of immune cells matters as well, as in hot tumors and responders to anti-PD-1, immune cells are adjacent to tumor cells, compared to non-responders whose TME is characterized immune-excluding phenotypes where immune cells are found in the stroma. In addition, immune-evading phenotypes with high immune cell infiltration but also upregulated immune checkpoints predict poor outcomes by single-cell RNA sequencing (20). High grade non-muscle-invasive bladder cancers start out with hot TME and gradually become more immune-attenuated with disease progression to muscle-invasive disease (21). Therefore, better understanding of TME dynamics may aid with maintaining hot TME throughout disease progression and preventing resistance to ICI. Recently, it was shown by spatial sequencing that immune cell niches characterized by tertiary lymphoid structures (TLS) and T cell-B cell coordinated immune responses are significantly enriched in responders to neoadjuvant therapy and improved prognosis in muscle-invasive disease (22) (**Figure 1**).

## 3 Immunotherapy in non-muscle invasive bladder cancer

### 3.1 Bacille Calmette-Guerin

Intravesical Bacillus Calmette–Guérin (BCG) represents one of the earliest and most enduring examples of successful cancer immunotherapy and has shaped the management of non–muscle invasive bladder cancer (NMIBC) for nearly five decades (23). The antitumor properties of BCG were first suggested in the mid-20th century through preclinical observations demonstrating enhanced resistance to tumor implantation in BCG-exposed animal models, followed by early clinical signals in hematologic malignancies, most notably the report by Mathe et al. in 1969 describing its activity in acute lymphoblastic leukemia (24). Building on these immunologic insights, Morales and colleagues first applied BCG intravesically in 1976, reporting a marked reduction in recurrence among patients with recurrent NMIBC treated after transurethral resection (25). These findings were rapidly validated in subsequent randomized controlled trials throughout the late 1970s and 1980s, which consistently demonstrated that intravesical BCG reduced tumor recurrence and delayed progression compared with surgical resection alone (26). Over time, accumulating evidence from large meta-analyses confirmed BCG’s superiority over intravesical chemotherapy, particularly when administered with maintenance therapy, firmly establishing BCG as the standard of care for intermediate- and high-risk NMIBC (27). Mechanistic studies further revealed that BCG exerts its antitumor effect through a complex, multifaceted immune response involving urothelial cytokine release, recruitment of innate immune cells, and activation of adaptive antitumor immunity, providing a biologic rationale for its durable clinical benefit. Together, these historical and biological foundations positioned BCG as a paradigm-defining immunotherapy and laid the groundwork for its widespread adoption in NMIBC, while also foreshadowing the challenges of immune heterogeneity and treatment resistance that continue to drive therapeutic innovation in this disease (28, 29).

### 3.2 Mechanisms

The antitumor activity of intravesical Bacillus Calmette–Guérin (BCG) in non–muscle invasive bladder cancer (NMIBC) reflects a multilayered immune process that integrates urothelial engagement, innate immune activation, and adaptive immune priming. Following installation, BCG binds to fibronectin on the urothelial surface via fibronectin attachment proteins, facilitating internalization into urothelial and tumor cells as well as professional antigen-presenting cells (30). This uptake triggers activation of pattern-recognition receptors, including toll-like receptors and NOD-like receptors, leading to downstream NF-κB signaling and robust secretion of proinflammatory cytokines and chemokines such as IL-1β, IL-6, IL-8, TNF-α, GM-CSF, and CXCL10 (28). These mediators orchestrate early recruitment of neutrophils, macrophages, dendritic cells, and natural killer cells, which contribute to tumor cell killing through cytotoxic granule release, reactive oxygen species, and antibody-dependent cellular cytotoxicity (29). With repeated exposure, antigen presentation and dendritic cell maturation promote a Th1-skewed adaptive immune response, characterized by expansion of CD4^+^ T helper cells, cytotoxic CD8^+^ T cells, and increased interferon-γ and IL-12 signaling, which are strongly associated with durable tumor control (28, 31). BCG has also been shown to modulate the tumor microenvironment by upregulating MHC class I and II expression on tumor cells, enhancing immune visibility, and altering immune checkpoint expression (32). In parallel, BCG may exert direct effects on tumor cells, including induction of apoptosis, cell-cycle arrest, and autophagy, although counter-regulatory mechanisms—such as inhibition of TNF-α–mediated apoptosis or expansion of regulatory T cells—may underlie primary resistance or eventual unresponsiveness (33, 34). Emerging data further supports a role for trained immunity, whereby epigenetic and metabolic reprogramming of innate immune cells following BCG exposure results in amplified and sustained antitumor responses upon re-challenge (35). Collectively, these diverse and overlapping mechanisms explain both the clinical efficacy of BCG and the heterogeneity of responses observed in NMIBC (36).

### 3.3 Dosing schedule

Current clinical practice guidelines emphasize that intravesical BCG remains the standard of care for patients with intermediate- and high-risk non–muscle invasive bladder cancer, with treatment intensity and duration tailored to individual risk profiles (37, 38). Across major guideline groups, including the American Urological Association/Society of Urologic Oncology (AUA/SUO), European Association of Urology (EAU), and National Comprehensive Cancer Network (NCCN), the backbone of BCG therapy is a 6-week induction course of weekly intravesical instillations administered after adequate healing from transurethral resection — typically beginning 2–4 weeks after resection and no later than 6 weeks to minimize systemic risks and optimize local immune activation (25, 26, 37, 38). Most guidelines endorse maintenance therapy, recognizing its role in reducing recurrence and progression; the classic SWOG (Lamm) schedule of three weekly instillations at 3, 6, 12, 18, 24, 30, and 36 months is widely used, with intermediate-risk patients generally receiving one year of maintenance and high-risk patients up to three years when tolerated (26, 27). The EAU also supports one-year maintenance for intermediate risk and up to three years for high risk, while acknowledging that extended maintenance must be balanced against side effects and supply constraints (37). During periods of BCG shortage, NCCN guidelines suggest prioritizing high-risk patients for both induction and early maintenance and allow dose sharing (e.g., splitting vials for induction) and dose reductions for maintenance, when necessary, although there is mixed evidence on the impact of reduced dosing on efficacy (39). Absolute contraindications include systemic BCG infection, severe immunosuppression, active tuberculosis, and pregnancy, while relative contraindications include unresolved urinary tract infection or compromised urothelial integrity at the time of planned instillation (26, 37). These guideline recommendations reflect an effort to maximize the durable, bladder-sparing benefit of BCG while mitigating toxicity and adapting to real-world constraints such as drug shortages and patient tolerability (23, 37).

### 3.4 Vaccines

Vaccine-based immunotherapy has increasingly been explored as an alternative or adjunct to Bacillus Calmette–Guérin (BCG) in non–muscle invasive bladder cancer (NMIBC), particularly for patients with BCG-unresponsive disease. Early work with peptide-based platforms showed promise, as demonstrated by Obara et al., who evaluated the subcutaneous vaccine S-288310 targeting DEPDC1 and MPHOSPH1 in previously untreated NMIBC and reported a 2-year recurrence-free survival of 74% when administered with BCG (40). Greater clinical momentum has been achieved with viral vector–based strategies.

Intravesical CG0070, a replication-competent oncolytic adenovirus designed to selectively replicate in retinoblastoma pathway–defective bladder cancer cells while promoting immunogenic cell death through GM-CSF expression, has shown meaningful activity in BCG-resistant NMIBC (36). In a phase II study, CG0070 monotherapy achieved a 6-month complete response (CR) rate of 47%, with responses enriched in patients with carcinoma *in situ* (CIS) and notably absent in those with pure T1 disease (36). Subsequent combination approaches appear to enhance efficacy further; in a study by Li et al., the addition of pembrolizumab to intravesical CG0070 resulted in a 3-month CR rate approaching 90% in BCG-resistant patients (41).

Not all vaccine strategies have translated into clinical benefit, however, as illustrated by PANVAC, which failed to improve recurrence-free or progression-free survival when added to BCG in the BCG-resistant setting (42). Unlike conventional therapeutic vaccines, nadofaragene firadenovec-vncg is a non-replicating adenoviral vector-based intravesical gene therapy that delivers the human interferon alfa-2b gene to urothelial cells, resulting in sustained local interferon production and enhancement of anti-tumor immune responses. The clinical relevance of intravesical gene therapy was firmly established with the U.S. Food and Drug Administration approval of Nadofaragene Firadenovec-vncg (Adstiladrin) in December 2022 for high-risk BCG-unresponsive NMIBC with CIS, with or without papillary disease. In the registrational study, a CR rate of 51% was observed using stringent criteria that included cystoscopy, biopsies, and urine cytology, with nearly half of responders maintaining remission for at least one year and a median duration of response of 9.7 months (43).

Earlier-generation systemic cancer vaccines, such as the NY-ESO-1 plasmid DNA vaccine, demonstrated biological activity but were limited by poor durability of T-cell responses, likely reflecting regulatory T-cell–mediated immune suppression and evolving epitope specificity over time (44). More recently, personalized neoantigen-based vaccination has renewed interest in systemic vaccine approaches; in a phase Ib study of the neoantigen peptide vaccine NEO-PV-01 combined with nivolumab, durable neoantigen-specific cytotoxic T-cell responses were observed in patients with advanced bladder cancer, with clinical responses correlating with expansion of memory CD8^+^ T-cell populations (45). Taken together, these studies suggest that while vaccine monotherapy has yielded variable results, intravesical viral platforms and rational combinations with immune checkpoint inhibition may offer a durable bladder-sparing strategy for selected patients, particularly those with CIS-predominant disease.

### 3.5 Immune checkpoint inhibitors in non-muscle-invasive bladder cancer

BCG is ineffective in 30-40% of patients, either due to a lack of initial response or later relapse. BCG-unresponsive patients are at a higher risk for progression to muscle-invasive bladder cancer, and treatment with radical cystectomy has been the main traditional option. Understanding the biological mechanisms of resistance is important for the discovery of rational salvage therapies. Lack of sufficient immune cell infiltration and high expression of immune checkpoint molecules in the TME are both seen in BCG-unresponsive tumors. In these patients, upregulation of PD-L1 by tumor cells and immune cells within the TME is a major mechanism of immune escape. Differences in innate immune response among individuals, such as the ability to activate TLR, may explain why some patients are less responsive to BCG (46).

The KEYNOTE-057 trial proved activity of PD-1 immune checkpoint blockade in BCG-unresponsive NMIBC. This Phase II trial tested pembrolizumab, an immune checkpoint inhibitor, in high-grade NMIBC patients with failure or relapse after BCG. The primary study endpoint was complete response (CR) rate at 3 months for carcinoma *in situ* (CIS) or papillary disease. Pembrolizumab achieved a 40.6% CR in the CIS cohort, which resulted in U.S. FDA approval as the first systemic immunotherapy for BCG-unresponsive NMIBC. Patients with an initial response experienced a long duration of control, with median duration not reached at the time of the primary study report (47).

The anti-PD-L1 immune checkpoint blocking antibody atezolizumab has been tested in NMIBC. The first Phase III trial of a checkpoint inhibitor in NMIBC, ALBAN, randomized high-risk treatment-naïve NMIBC patients to atezolizumab + BCG vs BCG alone, and measured improvement in event-free survival. However, the study arms performed nearly identically (HR 0.98; P = 0.9106), revealing that harnessing synergy between checkpoint inhibition and BCG is context- and agent-dependent. Patients treated with combination immunotherapy had increased risk of adverse events compared to BCG alone, supporting this as an appropriate strategy only with appropriate positive predictive biomarkers and in the absence of other, more effective treatments (48).

## 4 Immunotherapy in muscle-invasive bladder cancer

The treatment of muscle invasive bladder cancer has changed with immune checkpoint inhibitors (ICI) integration in all perioperative settings. Earlier decades showed neoadjuvant chemotherapy (NAC) followed by surgery significantly improved over all survival compared to surgery alone (49). The choice of chemotherapy regimens include gemcitabine and cisplatin (GC), standard methotrexate, vinblastine, doxorubicin, and cisplatin (MVAC) and dose-dense MVAC (ddMVAC) with ddMVAC showing higher efficacy rates at the expense of higher toxicities and complex logistics (50). Adjuvant nivolumab post-cystectomy as a standard of care is based on results of the Checkmate274 trial (51). Neoadjuvant immunotherapy (eg: as investigated in the PURE-1 trial) has also led to impressive results, with neoadjuvant pembrolizumab demonstrating high and long-lasting pathologic response rates even in patients not selected by the standard biomarkers (52). The profound results are being attained with chemo-immunotherapy combinations, as shown in the NIAGARA and KEYNOTE/EV trials, with significant improved pathological complete response, event-free survival, and overall survival in cisplatin eligible as well as cisplatin ineligible cohorts (53). Lastly, organ preservation strategies are evolving with neoadjuvant immunotherapy, as clinical complete response to neoadjuvant immunotherapy might open the possibility for bladder-sparing treatments (54) (**Table 1**).

**Table 1:** Perioperative immunotherapy outcomes in muscle-invasive bladder cancer.

Trial	Setting & population	Treatment & comparator	Outcomes
CheckMate 274 (51)	Adjuvant; high-risk MIBC post-cystectomy (pT3–T4 or N+)	Nivolumab vs placebo	DFS: 25.6 vs 8.5 mo (HR 0.71); OS trend favoring nivolumab
PURE-01 (56)	Neoadjuvant; cT2–T4a N0–N1 MIBC	Pembrolizumab (single arm)	pCR: 42%; responses observed regardless of PD-L1 status
NURE-Combo (57)	Neoadjuvant; variant histology MIBC	Pembrolizumab-based (single-arm)	~38% pCR in squamous differentiation
aMVAC + Pembrolizumab (58)	Neoadjuvant; non-urothelial MIBC	Chemotherapy + pembrolizumab (single arm)	Higher response rates in squamous differentiation
NIAGARA (59)	Perioperative; cT2–T4a N0–N1 (cisplatin-eligible)	Durvalumab + GC → surgery → durvalumab vs GC → surgery	pCR: 37.3% vs 27.5%; EFS HR 0.68; OS HR 0.75
KEYNOTE-905/EV-303 (60)	Perioperative; cisplatin-ineligible MIBC	Enfortumab vedotin + pembrolizumab vs surgery alone	pCR: 57.1% vs 8.6%; EFS HR 0.40; OS HR 0.50
KEYNOTE-B15/EV-304 (61)	Perioperative; cisplatin-eligible MIBC	Enfortumab vedotin + pembrolizumab vs GC chemotherapy	pCR: 55.8% vs 32.5%; EFS HR 0.53; OS HR 0.65
HCRN GU 16–257 (54)	Bladder-sparing; MIBC with clinical complete response	GC + nivolumab → nivolumab maintenance (single arm)	cCR: 43%; >80% 2-year bladder-intact metastasis-free survival

MIBC, muscle-invasive bladder cancer; GC, gemcitabine + cisplatin; pCR, pathologic complete response; DFS, disease-free survival; HR, Hazard ratio; EFS, event-free survival; OS, overall survival; cCR, clinical complete response; PDL1, Programmed cell death ligand 1.

### 4.1 Adjuvant ICI

The CheckMate 274 trial represents the pivotal evidence establishing adjuvant nivolumab as FDA-approved standard of care for high-risk MIBC. This phase 3, randomized, double-blind trial enrolled 709 patients with pathological stage pT3-T4 or node-positive disease after radical cystectomy and randomized them 1:1 to receive nivolumab 240 mg intravenously every 2 weeks or placebo for up to 1 year. The primary endpoint was disease-free survival in both the intention-to-treat (ITT) population and in patients with tumor PD-L1 expression ≥1%. Results demonstrated a disease-free survival hazard ratio of 0.71 (95% CI: 0.55-0.90) in the ITT population, with median disease-free survival of 25.6 months in the nivolumab group versus 8.5 months in the placebo group (51).

Extended follow-up with 36.1-month median follow-up confirmed durability of benefit and provided the first overall survival data. The overall survival hazard ratio favored nivolumab with a value of 0.76 (95% CI: 0.61-0.96) in the ITT population and 0.56 (95% CI: 0.36-0.86) in patients with PD-L1 ≥1% expression. In the MIBC subgroup (n=560), all MIBC patients (HR 0.70) and those with PD-L1 ≥1% (HR 0.48) showed overall survival benefit regardless of prior neoadjuvant chemotherapy status. Grade 3–4 treatment-related adverse events occurred in 18% of nivolumab-treated patients versus 8% in the placebo group, with immune-related adverse events generally manageable with standard corticosteroid therapy. These results support adjuvant nivolumab as a standard of care for all eligible patients with high-risk MIBC (55).

### 4.2 Neo adjuvant ICI

PURE-01 investigated pembrolizumab as neoadjuvant monotherapy. This open-label, single-arm, phase 2 trial enrolled 155 patients with T2-T4a N0-N1 MIBC and administered four cycles of pembrolizumab 200 mg intravenously every 3 weeks prior to radical cystectomy. Pathological complete response (ypT0N0) was achieved in 42% of patients, with an additional 12% achieving partial response (56).

Median follow-up of 39 months demonstrated event-free survival of 74.4% and overall survival of 83.8% (95% CI: 77.8-90.2). Patients with PD-L1 combined positive score ≥50% showed improved event-free survival versus those with lower scores (89.8% vs 59.7%, p=0.0013), but pathological complete response rates of 25-35% were still observed in PD-L1-negative patients (52).

Importantly, outcomes in patients with non-urothelial histologies were also analyzed in detail, and responses were shown to vary dramatically by histology. In the NURE-Combo trial, patients with squamous cell carcinoma differentiation achieved a pathological complete response rate of 38%, although much higher rates were achievable (57). In the aMVAC-pembrolizumab trial of non-UC MIBC, patients with squamous differentiation had the highest response rates (58).

### 4.3 ICI combinations

A newly published, randomized phase 3 trial, NIAGARA, provides the highest level of evidence to recommend chemo-immunotherapy combinations in the perioperative management of MIBC. In all, 1,063 cisplatin-eligible patients with cT2-T4a N0-N1 MIBC were randomized 1:1 to receive either neoadjuvant durvalumab (1500 mg intravenously every 3 weeks for 4 cycles) plus gemcitabine-cisplatin (GC) followed by radical cystectomy with adjuvant durvalumab for 8 cycles, or neoadjuvant GC followed by radical cystectomy (59).

Durvalumab plus chemotherapy achieved a 37.3% complete response compared to 27.5% in the chemotherapy-alone group. More importantly, event-free survival at 24-months was 67.8% in the durvalumab group compared to 59.8% in the comparison group (hazard ratio, 0.68; 95% CI, 0.56 to 0.82). Overall survival at 24 months was 82.2% in the durvalumab group and 75.2% in the comparison group (hazard ratio, 0.75; 95% CI, 0.59 to 0.93) Grade 3–4 treatment-related adverse events occurred in 40.6% of the durvalumab group and 40.9% of the comparison group.

The phase 3 KEYNOTE-905/EV-303 trial confirmed the role of perioperative EV plus pembrolizumab in predominantly cisplatin-ineligible MIBC (n=344), demonstrating improvements over surgery alone in event-free survival (74.7% vs 39.4%; hazard ratio, 0.40; 95% CI, 0.28 to 0.57; P&lt;0.001) and overall survival (79.7% vs 63.1%; hazard ratio, 0.50; 95% CI, 0.33 to 0.74; P&lt;0.001) at 2-year follow-up. Most dramatically, EV-pembrolizumab achieved a pathological complete response rate of 57.1% compared to 8.6% in the surgery-alone group, an absolute difference of 48.3 percentage points (95% CI, 39.5 to 56.5; P&lt;0.001) (60).

The KEYNOTE-B15/EV-304 trial extended this paradigm to cisplatin-eligible patients (n=808), demonstrating that EV plus pembrolizumab could challenge standard cisplatin-based chemotherapy. This phase 3 randomized trial compared neoadjuvant and adjuvant EV plus pembrolizumab (4 cycles neoadjuvant, followed by 5 cycles adjuvant EV plus 13 cycles adjuvant pembrolizumab) versus standard neoadjuvant gemcitabine-cisplatin (GC) followed by observation. EV plus pembrolizumab achieved 24-month event-free survival of 79.4% versus 66.2% (hazard ratio, 0.53; 95% CI, 0.41 to 0.70; P = 0.0001), 24-month overall survival of 86.9% versus 81.3% (hazard ratio, 0.65; 95% CI, 0.48 to 0.89; P = 0.0029), and pathological complete response of 55.8% versus 32.5% (estimated difference, 23.4%; 95% CI, 16.7 to 29.8) (61).

**Figure 2:**
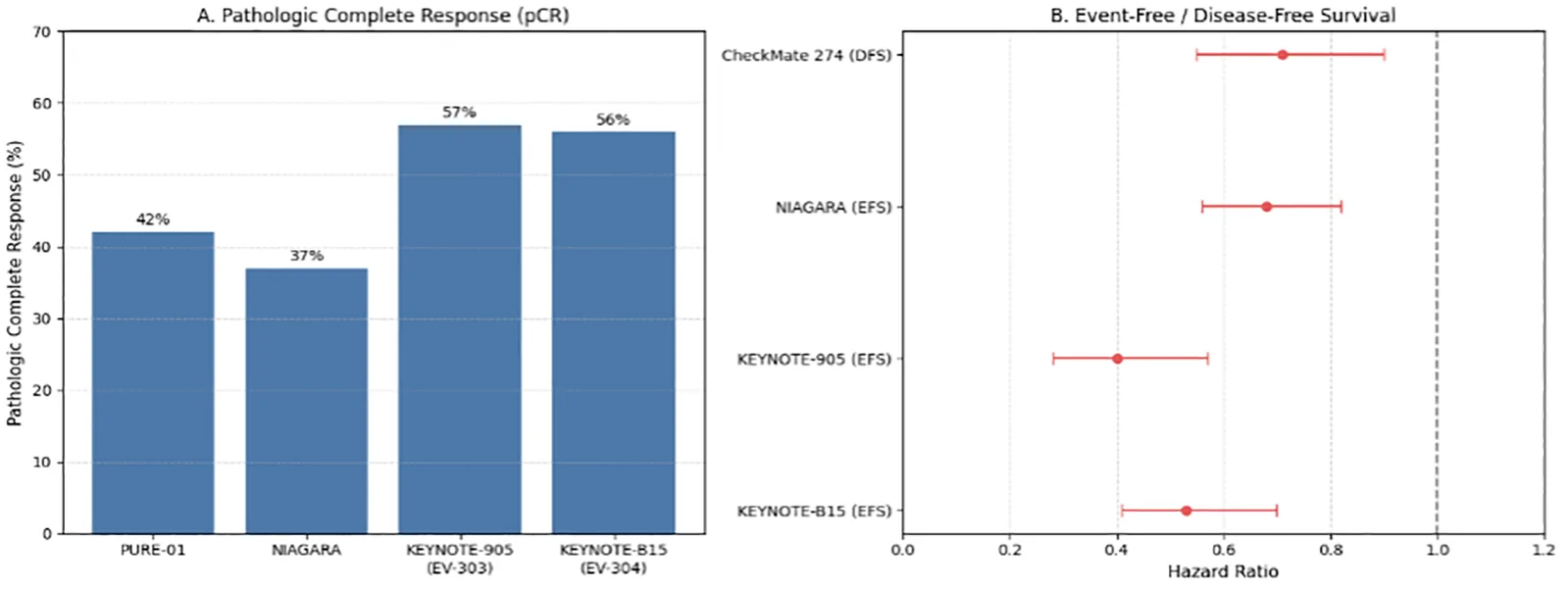
Perioperative immunotherapy outcomes in muscle-invasive bladder cancer.

Taken together, these phase III results are practice-changing and strongly support enfortumab vedotin plus pembrolizumab as an important emerging standard in the perioperative management of bladder cancer, although longer-term follow-up and real-world experience will further clarify its position relative to other treatment approaches (**Figure 2**).

An indirect comparison of KEYNOTE-905/EV-303 to the PURE-01 pembrolizumab monotherapy trial was performed using patient-level data. Compared to pembrolizumab monotherapy, EV plus pembrolizumab improved pCR (57.1% vs 39.5%), but not event-free survival (hazard ratio, 0.75; 95% CI, 0.49 to 1.14; p=0.181), suggesting both a role for the antibody-drug conjugate as well as adjuvant timing of therapy. Of note, grade ≥3 treatment-related adverse events were reported in 75.7% of patients who received EV plus pembrolizumab, as compared with 67.2% of those who received chemotherapy, and the most common grade ≥3 drug-related adverse event was skin reactions for both EV (14.1%) and pembrolizumab (13.9%), which are generally manageable (62).

### 4.4 Role of immunotherapy for bladder sparing strategies

Concurrent chemoradiation with 5fu/mitomycin or cisplatin is the standard for patient who are eligible for bladder sparing. It was evaluated in the BC2001 by adding concurrent chemotherapy with 5-fluorouracil and mitomycin to radiotherapy in patients with muscle-invasive bladder cancer which significantly improved 10-year locoregional disease-free survival compared with radiotherapy alone (hazard ratio 0.61), with lower rates of invasive locoregional recurrence (18% vs 32%) and higher rates of locoregional control and 5-year overall survival rates (48% vs 35%) but similar long-term overall survival rates (63).

Stringent evaluation of clinical complete response with multi-modal assessment is a promising approach. Studies have shown that patients with negative ct DNA had better survival compared to ct DNA positive patients (64). Neoadjuvant chemotherapy, chemo immunotherapy combinations resulted in pathologic CR of around 40% and had led to explore if neoadjuvant chemo immunotherapy as an alternative to definitive chemoradiation for a bladder sparing approach (65, 66).

In HCRN GU 16-257, patients with clinical complete response (including a normal cytology, no evidence of disease on imaging, and cT0/Ta on cystoscopy and biopsy) after treatment with 4 cycles of gemcitabine-cisplatin and nivolumab were candidates for bladder preservation with surveillance and three more cycles of nivolumab. Clinical complete response occurred in 43% of patients (33/76 enrolled).

These patients did not undergo cystectomy and instead received nivolumab consolidation plus intensive surveillance. Two-year bladder-intact metastasis-free survival was over 80%, suggesting this stringent approach to identification of complete responders is a safe path to avoid cystectomy in the right population. Baseline tumor mutational burden ≥10 mut/MB was significantly associated with clinical complete response (p=0.01) in this trial (54). Clinical trials are now looking to see if multimodal assessment with ct DNA, Urinary ct DNA, cystoscopy and imaging can identify muscle invasive bladder cancer patients treated with neoadjuvant chemo immunotherapy combinations and can omit chemo radiation for bladder sparing.

### 4.5 Immunotherapy in metastatic bladder cancer

Historically metastatic bladder cancer has been treated using platinum based combination chemotherapy regimens (67, 68). The recent phase III EV-302 trial was practice-changing, showing that enfortumab vedotin (EV) plus pembrolizumab improves both progression-free survival (PFS, 12.5 vs. 6.3 months, HR 0.45) and overall survival (OS, 31.5 vs. 16.1 months, HR 0.47) compared with platinum-based chemotherapy, making it the new standard-of-care for all patients with mUC (69). Coinciding with these results, the CheckMate-901 trial found that the addition of nivolumab to cisplatin and gemcitabine also improved survival (OS 21.7 vs. 18.9 months, HR, 0.78), further supporting the benefit of immune checkpoint inhibition in the front-line setting, although uptake may be limited given the better results achieved with EV plus pembrolizumab. Importantly, while both trials established chemoimmunotherapy-based approaches as superior to chemotherapy alone, EV-302 demonstrated substantially larger improvements in both PFS and OS across a broader patient population, including cisplatin-ineligible patients, supporting its emergence as the preferred first-line regimen for most patients with metastatic urothelial carcinoma (70). Maintenance avelumab for patients without progression after chemotherapy was established by the JAVELIN Bladder 100 trial, and KEYNOTE-052 established single-agent pembrolizumab as an option for patients who are ineligible for any platinum chemotherapy, however the use of maintenance or single-agent ICI in the first-line setting is likely to decline with immune-based combinations being widely adopted. Collectively, these studies illustrate the rapid evolution of first-line management from sequential chemotherapy followed by immunotherapy to upfront combination strategies that integrate immune checkpoint inhibition earlier in the disease course (71, 72) (**Table 2**).

**Table 2:** Summary of first line immunotherapy trials in metastatic bladder cancer.

Trial	Regimen/comparator	Population (n)	Primary endpoints	Key results
EV-302 (NCT04223856) (69)	Enfortumab vedotin + Pembrolizumab vs. Gemcitabine + Platinum (cisplatin/carboplatin)	886	PFS, OS	- PFS: 12.5 vs. 6.3 mo (HR 0.45, p<0.00001) - OS: 31.5 vs. 16.1 mo (HR 0.47, p<0.00001) - ORR: 67.7% vs. 44.4%
CheckMate-901 (NCT03036098) (70)	Nivolumab + Gemcitabine + Cisplatin vs. Gemcitabine + Cisplatin	608	PFS, OS	- PFS: 7.9 vs. 7.6 mo (HR 0.72, p=0.001) - OS: 21.7 vs. 18.9 mo (HR 0.78, p=0.02) - ORR: 57.6% vs. 43.1%
JAVELIN Bladder 100 (NCT02603432) (71)	Maintenance Avelumab vs. Best Supportive Care after first-line Platinum Chemo	700	OS	- OS: 21.4 vs. 14.3 mo (HR 0.69, p=0.001) - Benefit independent of PD-L1 status
KEYNOTE-052 (NCT02335424) (72)	Pembrolizumab monotherapy (single arm)	370	ORR (Primary), OS	- ORR: 24% (95% CI 20–29) - OS: 11.3 mo
KEYNOTE-361 (NCT02853305)	Pembrolizumab ± Chemotherapy vs. Chemotherapy	1010	PFS, OS	- No significant OS/PFS benefit for pembrolizumab arms

PFS, Progression-free survival; HR, Hazard ratio; OS, overall survival; ORR, Over all response rate; PDL1, Programmed cell death ligand 1.

Prospective randomized trials remain the highest level of evidence for establishing efficacy, but real-world data are increasingly important for understanding how these therapies perform in routine practice. In advanced urothelial carcinoma, emerging observational cohorts suggest that enfortumab vedotin plus pembrolizumab retains meaningful clinical activity and manageable toxicity outside the trial setting, with reported outcomes that are broadly consistent with phase III experience. Real-world studies also help contextualize treatment selection, tolerability, and duration of therapy in older and more comorbid patients who are often underrepresented in clinical trials. Together, these data support the external validity of the pivotal studies while highlighting the need for continued follow-up and broader post-approval experience (101).

Following progression after platinum-based chemotherapy, three immune checkpoint inhibitors (ICI) (pembrolizumab, nivolumab, and avelumab) were approved by the FDA for use in the second-line metastatic setting regardless of PD-1/PD-L1 status. Among these, only pembrolizumab has demonstrated a significant overall survival benefit; in the phase III KEYNOTE-045 trial versus chemotherapy in the second-line setting, pembrolizumab improved OS (10.3 vs 7.4 months, HR 0.73, p = 0.002) and objective response rate (21.1% vs 11.4%) with fewer treatment-related adverse events (73). Nivolumab was tested in the single-arm CheckMate 275 study with an ORR of 19.6% and granted accelerated approval based on the durability of response, but without a confirmatory OS study (74).Avelumab was granted approval following the JAVELIN Solid Tumor trial (ORR 16.5%) with modest activity and limited toxicity, but again without a confirmatory phase III study (75). These differences in trial design are clinically important, as pembrolizumab remains the only agent supported by level I evidence demonstrating an overall survival advantage, whereas approvals for nivolumab and avelumab were based primarily on durable response rates from single-arm studies. Both atezolizumab and durvalumab were withdrawn from the second-line setting when they failed to improve OS in phase III trials (76, 77). In the new era of first-line pembrolizumab plus enfortumab vedotin, the relevance of any single-agent ICI in the second-line or greater setting is unclear. Future treatment sequencing will likely depend on identifying effective therapies following progression on EV plus pembrolizumab, an area for which prospective evidence remains limited (**Table 3**).

**Table 3:** Summary of later-line immunotherapy trials in metastatic bladder cancer.

Trial	Agent/comparator	Population (n)	Primary endpoint(s)	Key results
KEYNOTE-045 (NCT02256436) (73)	Pembrolizumab vs. Chemotherapy (paclitaxel, docetaxel, or vinflunine)	542	OS, PFS	- OS: 10.3 vs. 7.4 mo (HR 0.73, p = 0.002) - ORR: 21.1% vs. 11.4% - PFS: no difference - Fewer treatment-related AEs (60.9% vs. 90.2%)
CheckMate-275 (NCT02387996) (74)	Nivolumab (single arm)	270	ORR	- ORR: 19.6% (95% CI 15.0–24.9%) - Grade 3–4 AEs: 18%
JAVELIN Solid Tumor (NCT01772004) (75)	Avelumab (single arm)	161 (pooled)	ORR	- ORR: 16.5% (95% CI 11.9–22.4%) - CR: 4.1% - PR: 12.4% - Serious AEs: 8%, including 1 death from pneumonitis
IMvigor211 (NCT02302807) (76)	Atezolizumab vs. Chemotherapy	931	OS	- No OS benefit vs. chemotherapy
DANUBE (NCT02516241) (77)	Durvalumab ± Tremelimumab vs. Chemotherapy	1032	OS (PD-L1 high and ITT)	- No OS improvement in either population

PFS, Progression-free survival; HR, Hazard ratio; OS, overall survival; ORR, Over all response rate; PDL1, Programmed cell death ligand 1; ITT, Intention to treat.

## 5 Biomarker integration and artificial intelligence

PD-L1 expression is one of the most studied biomarkers for immune checkpoint inhibitor response in MIBC (78). In PURE-01, patients with a high PD-L1 expression (combined positive score ≥50%) had improved pathological complete response rates and event-free survival than those with lower PD-L1 expression (89.8% vs 59.7%, p=0.0013). However, importantly, patients with PD-L1 negative tumors still had significant pathologic complete response rates ranging from 25-35%, suggesting that PD-L1 should not be used to exclude patients from immunotherapy treatment (52).

The NIAGARA trial likewise demonstrated that durvalumab benefit was observed in both PD-L1 negative and positive subgroups, without evidence of substantial heterogeneity in treatment effect, reinforcing PD-L1 as more of a prognostic marker. Spatial and temporal heterogeneity, as well as inter-assay differences between different antibody clones, limits utility of PD-L1 as a single biomarker (59).

Tumor mutational burden (TMB), number of nonsynonymous mutations per exome or megabase of exome/genome sequencing, is an FDA-approved biomarker for immune checkpoint inhibitor response in various cancer types (79). In bladder cancers, TMB predicts ICI response in the metastatic setting. However, TMB predicts ICI response well in tumors with favorable TME with high CD8+ T cell infiltration and M1 macrophage population, while it does not predict response in those with TME characterized by abundant M2 macrophages and high stromal content (2, 3). Moreover, TMB needs to be adjusted for clonality, as clonal TMB but not subclonal TMB predicts immune checkpoint inhibitor response (80).

Circulating tumor DNA is another very promising biomarker for MIBC, both prognostically and predictively (53). Dynamic ctDNA response is correlated to pathological response with clearance of ctDNA from baseline to pre-operative RC was achieved in 41% of the durvalumab arm and 31% of the chemotherapy only arm, demonstrating additional benefit to the combination of immunotherapy plus chemotherapy. In pre-operative ctDNA-positive patients, the non-pCR rate was 97%. ctDNA easily enables dynamic assessment of overall treatment response and can detect minimal residual disease well in advance of clinical or radiographic recurrence (81). IMVigour011 further demonstrated the biomarker capabilities of ctDNA in guiding the decision to use adjuvant immunotherapy among those who were ctDNA positive after surgery for MIBC and thereby significantly improving disease free survival (9.9m vs 4.8m; p=0.005) and overall survival (32.8m vs 21.1m; p=0.01) (64). RETAIN trials on the other hand demonstrated higher metastasis-free survival (85% at 2 years) among ctDNA-negative patients, thereby avoiding life-altering radical cystectomy, while revealing a 10.7-fold higher risk of metastasis among the ctDNA positive cohort (82).

Taken together, these biomarkers represent different facets of tumor biology but have distinct clinical limitations. PD-L1 expression is inexpensive and widely available but suffers from substantial spatial, temporal, and assay-related variability, limiting its predictive value. TMB provides a broader measure of tumor immunogenicity but is influenced by the surrounding tumor immune microenvironment and lacks standardized thresholds and analytical methods across sequencing platforms. Among currently investigated biomarkers, ctDNA appears particularly promising because it enables dynamic assessment of treatment response and minimal residual disease; however, prospective validation and assay standardization remain necessary before widespread clinical implementation. However, despite its promise, widespread implementation remains limited by assay standardization, cost, availability, and the need for prospective validation demonstrating improved patient outcomes when treatment decisions are guided by ctDNA. Consequently, no single biomarker currently possesses sufficient predictive accuracy to guide routine immunotherapy selection in MIBC, highlighting the need for integrated biomarker approaches.

Artificial intelligence will likely enable precision management of MIBC in the future (83). AI and machine learning (ML) algorithms are particularly adept at integrating heterogenous multi-omics data sets (genomics, transcriptomics, proteomics, epigenomics, metabolomics, microbiomics, and radiomics among others) to create deployable composite biomarkers and to maintain biological explainability using methods such as SHAP and GradCAM (84). Rather than replacing existing biomarkers, AI offers the opportunity to integrate multiple complementary biomarkers into composite predictive models that better capture the complexity of tumor biology and the tumor immune microenvironment.

Multi-institutional studies have shown that integrating multi-omics data with clinical and radiologic variables using AI greatly improves modeling response and survival outcomes above and beyond standard biomarker approaches (85). In the perioperative MIBC space, multi-omics analysis has revealed clusters of tumors with different oncogenic signaling pathways and highly variable levels of signaling from the microenvironment. Specifically, proteogenomic analysis reveals that FGFR3 mutant tumors have increased glycolysis, extracellular matrix (ECM) remodeling, and lowered immune regulatory signaling, with decreased CD8+ T cell infiltration. Non-responders to BCG are correlated with cancer-associated fibroblast signatures, decreased infiltration of CD8+ T cells, and high expression of the ECM modulator PLOD1. Therefore, combining immunotherapy with approaches that disrupt cancer-PLOD1 interactions with CAFs hold promise to overcome resistance to BCG and immune checkpoint blockade (86).

SWI/SNF chromatin-remodeling complex alterations are an example of a specific genotype which is predictive of response to immune checkpoint blockade; they occur in 42.8% (626/1463) of urothelial bladder cancers and have high overall predictive accuracy (C-index > 0.75) using machine learning models integrating multi-omics data compared to a single biomarker; machine learning modeling achieved high accuracy in predicting patient overall survival from ICI immunotherapy (0.776 for all patients on the test set; AUC in the SWI/SNF-mutant group on the test set reached 0.909) (87). Ultimately, machine learning classifiers that integrate many omics layers with radiomic and clinical variables will be necessary to accurately predict response to neoadjuvant immune checkpoint blockade.

## 6 Emerging immunotherapeutic platforms

Beyond the use of checkpoint blockade and intravesical vaccines, there are many emerging immunotherapeutic modalities actively being explored to potentiate response and delay resistance in bladder cancer by targeting different aspects of the tumor microenvironment, and harnessing technological advancements in cellular engineering, metabolism, and delivery. The ability to combine multiple immune-manipulating mechanisms at the same time may provide an advantage over single strategy therapies (46).

CAR-T therapy has been successful in targeting antigens in hematologic cancers and is transitioning to solid tumor space with some additional challenges (88). Next-generation CAR-T cells include both antigen-specific targeting, production of endogenous growth factors or cytokines, and knockout of inhibitory markers such as PD-1 (89).

On the other hand, CAR-NK cell therapy is in development with the advantages of rapid killing, lower toxicities and reduced risk of genomic remodeling(cytokine storm) (90). PD-L1 targeting CAR-NK cells (t-haNK) show strong efficacy in bladder cancer and combinatorial therapy with both IL-15 super agonist and anti-PD-1 (TriKE) leads to improved success. The parent CAR-NK cell line includes an IL-15 gene to enhance T-cell and NK cell expansion, allowing for combinations of stimuli at the same time (91).

IL-15 is a potent immunostimulant through activation of NK cell, NKT cell, and memory CD8+ T cell expansion, whereas IL-2 primarily expands immunosuppressive Tregs (92). IL-15 super agonists are under development for use in monotherapy or in combination strategies. In particular, bi- and tri-specific molecules can provide PD-1 and TIGIT blocking antibodies alongside activation of CD8+ T cells through IL-15 and epigenetic markers. For example, bifunctional immunity reprogramming T cell engager (PD-1/TIGIT blocking and IL15 trigger for metabolism and function) can change the phenotype of CD8+ T cells in mouse models, and demonstrate increased efficacy compared to combination CIP therapy (92).

IDO-1 is a tryptophan-catabolizing enzyme involved in regulating adaptive immune responses. By consuming tryptophan, it promotes Treg induction, T cell anergy, and CD8+ T cell exhaustion. IDO-1 expression is increased in bladder tumors and has a negative effect on prognosis (93). IDO-1 inhibitors lift the metabolic suppression of T cell proliferation to enhance the cytotoxic effect of checkpoint blockade. A clinical trial of pembrolizumab and the IDO-1 inhibitor epacadostat is ongoing in BCG-R NMIBC, and more studies will investigate this combination strategy in the future (94).

In addition to intravesical therapy with oncolytic viral DNA vectors CG0070 and nadofaragene firadenovec, oncolytic RNA viruses can be engineered for high replication in tumor cells and immunogenic cell death in bladder cancer (95). These increase expression of PD-L1 in the TME and proinflammatory cytokines, which sensitize tumors to checkpoint inhibitors and improve immune infiltration. Immunogenic cell death inducers, in particular ferroptosis inducers, are under development for use in combination with checkpoint blockade. These therapies can improve the efficacy of PD-1/PDL-1 inhibitors by triggering CD8+ T cell-mediated immunogenic death and recruitment of proinflammatory myeloid cells (96).

Dendritic Cell (DC) vaccines using tumor lysates or neoantigen peptides (or mRNA) are showing promise in combination with checkpoint blockade. Neoantigen-loaded DC vaccine NEO-PV-01 with anti-PD-1 antibody nivolumab elicits durable neoantigen-specific CD8+ T cell responses in melanoma, non-small cell lung cancer, and bladder cancer. Patient responses are correlated with expansion of memory CD8+ T cells. The future is bright for DC and neoantigen vaccination, but patient-specific personalization will be necessary for optimal responses (97).

As outlined in many of the examples above, combined strategies for immunotherapeutic manipulation of the bladder TME will be the way of the future (98). Some important spaces of development include (i) ICB with another immunotherapeutic or second ICB targeting a different negative costimulatory mAb (i.e. anti-LAG-3 with anti-PD-1), (ii) TGFb1 targeting with anti-PD-1, (iii) antibody drug conjugates with ICB for increased immunogenicity and persistence and (iv) additional metabolism-targeting drugs for adenosine, lactate, and tryptophan (99). multi-target immunomodulatory nanomaterials can carry many drugs (agonists and antagonists) at the same time in better delivery systems that react to different stimuli (100).

A critical challenge to the successful implementation of these therapeutic advances in combination strategy will be identifying which patients are most likely to benefit from each therapy. Biomarker selection will move beyond PD-L1, TMB and standard immunohistochemistry to include prediction of the immune and metabolic microenvironment before and during early treatment. Algorithms based on patient characteristics will integrate genomics, transcriptomics, and proteomics to select those most likely to respond to a given therapy.

## 7 Conclusion

Immunotherapy in bladder cancer has made great strides, from the decade’s old standard of BCG to a wider variety of immunotherapy combination approaches available in multiple disease contexts. However, durable response rates are still limited to only a small fraction of patients due in part to the tremendous barrier of overcoming a heterogeneous tumor microenvironment. The next chapter in bladder cancer immunotherapy will require better patient selection and thoughtful sequencing of therapies. Combination genomic-transcriptomic-spatial immune biomarkers may better identify optimal candidates for immunotherapy. Novel immune-based therapeutic approaches such as adoptive cell therapy, microbiome modulation, and oncolytic viruses may overcome innate and adaptive resistance to currently available immunotherapies. Linking scientific and clinical investigation will pave the way toward further improving immunotherapy response and providing durable benefits to a wider proportion of patients with bladder cancer.

## References

[1] Zhang L-Y Wang P Wang Y-B He Z-Q . Global, regional, and national burden of bladder cancer, 1990-2019: an age-period-cohort analysis based on the Global Burden of Disease 2019 study. Public Health. (2024) 236:193–203. doi: 10.1016/j.puhe.2024.07.027 39265377

[2] Liao W Zhou X Lin H Feng Z Chen X Chen Y . Interplay between tumor mutation burden and the tumor microenvironment predicts the prognosis of pan-cancer anti-PD-1/PD-L1 therapy. Front Immunol. (2025) 16:1557461. doi: 10.3389/fimmu.2025.1557461 40777041 PMC12328289

[3] Sun S Liu L Zhang J Sun L Shu W Yang Z . The role of neoantigens and tumor mutational burden in cancer immunotherapy: advances, mechanisms, and perspectives. J Hematol Oncol. (2025) 18:84. doi: 10.1186/s13045-025-01732-z 40898324 PMC12406617

[4] Filho AM Briganti A Jemal A Bray F . Bladder cancer incidence and mortality: a global overview and recent trends. Eur Urol. (2025) 89(5):426–36. doi: 10.1016/j.eururo.2025.12.011 41421927

[5] Strandgaard T Andreasen TG Divita TJ Salminen L Houghton S Thorsen K . Abstract 769: Spatial proteomics and transcriptomics reveal an altered immune cell landscape in bladder cancer patients unresponsive to BCG treatment. Cancer Res. (2025) 85:769. doi: 10.1158/1538-7445.AM2025-769 36230740

[6] Miyake M Tatsumi Y Gotoh D Ohnishi S Owari T Iida K . Regulatory T cells and tumor-associated macrophages in the tumor microenvironment in non-muscle invasive bladder cancer treated with intravesical Bacille Calmette-Guérin: a long-term follow-up study of a Japanese cohort. Int J Mol Sci. (2017) 18(10):2186. doi: 10.3390/ijms18102186 29048388 PMC5666867

[7] Di Spirito A Balkhi S Vivona V Mortara L . Key immune cells and their crosstalk in the tumor microenvironment of bladder cancer: insights for innovative therapies. Explor Target Antitumor Ther. (2025) 6:1002304. doi: 10.37349/etat.2025.1002304 40177538 PMC11964778

[8] Pfannstiel C Strissel PL Chiappinelli KB Sikic D Wach S Wirtz RM . The tumor immune microenvironment drives a prognostic relevance that correlates with bladder cancer subtypes. Cancer Immunol Res. (2019) 7:923–38. doi: 10.1158/2326-6066.CIR-18-0758 30988029

[9] Fan C Zhu W Chen Y Zhu W Ding J . Cancer-associated fibroblasts: origin, classification, tumorigenicity, and targeting for cancer therapy. MedComm (Beijing). (2025) 6:e70415. doi: 10.1002/mco2.70415 41164803 PMC12559857

[10] Hussain T Saha D Chakraborty A . Next-generation immune checkpoint inhibitors in cancer: mechanisms, current clinical advances, and future directions. J Cancer Immunol (Wilmington). (2025) 7:144. doi: 10.33696/cancerimmunol.7.113

[11] Liu Q Guan Y Li S . Programmed death receptor (PD-)1/PD-ligand (L)1 in urological cancers : the “all-around warrior” in immunotherapy. Mol Cancer. (2024) 23:183. doi: 10.1186/s12943-024-02095-8 39223527 PMC11367915

[12] Cui C Zhang H Yang C Yin M Teng X Yang M . Inhibition of JNK signaling overcomes cancer-associated fibroblast-mediated immunosuppression and enhances the efficacy of immunotherapy in bladder cancer. Cancer Res. (2024) 84:4199–213. doi: 10.1158/0008-5472.CAN-24-0940 39292817

[13] Wang X He J Ding G Tang Y Wang Q . Overcoming resistance to PD-1 and CTLA-4 blockade mechanisms and therapeutic strategies. Front Immunol. (2025) 16:1688699. doi: 10.3389/fimmu.2025.1688699 41112253 PMC12531160

[14] Leone RD Emens LA . Targeting adenosine for cancer immunotherapy. J Immunother Cancer. (2018) 6:57. doi: 10.1186/s40425-018-0360-8 29914571 PMC6006764

[15] Liu Z Li Y Cao J Qiu Y Yu K Deng S . The role of macrophages in cancer: from basic research to clinical applications. MedComm (Beijing). (2026) 7:e70547. doi: 10.1002/mco2.70547 41426206 PMC12717452

[16] Semenza GL . Intratumoral hypoxia and mechanisms of immune evasion mediated by hypoxia-inducible factors. Physiol (Bethesda). (2021) 36:73–83. doi: 10.1152/physiol.00034.2020 33595388

[17] Marbaniang C Kma L Sharan RN . Immunometabolism in the tumor microenvironment: dual role in modulating anti-tumor immunity and tumor progression. Asian Pac J Cancer Prev. (2025) 26:3881–93. doi: 10.31557/APJCP.2025.26.11.3881 41312912 PMC12927519

[18] Sang M Ge J Ge J Tang G Wang Q Wu J . Immune regulatory genes impact the hot/cold tumor microenvironment, affecting cancer treatment and patient outcomes. Front Immunol. (2025) 15. doi: 10.3389/fimmu.2024.1382842 39911580 PMC11794490

[19] Nagumo Y Ye X Shi T Mathis BJ Sakurai T Nishiyama H . Revealing intra-group immunotherapy response heterogeneity in metastatic urothelial carcinoma through interpretable feature extraction and spectral clustering. Front Immunol. (2026) 16. doi: 10.3389/fimmu.2025.1629001 41567195 PMC12816215

[20] Fazili A Gullapalli K Roman Souza G Hatoum F Miller J Kim Y . Geospatial and cell density analysis using multiplex immunofluorescence reveals an important role of clustering patterns of immunosuppressive macrophages in survival outcomes of penile squamous cell carcinoma. Cancers (Basel). (2026) 18:257. doi: 10.3390/cancers18020257 41595176 PMC12839248

[21] Li J Jiang Y Ma M Wang L Jing M Yang Z . Epithelial cell diversity and immune remodeling in bladder cancer progression: insights from single-cell transcriptomics. J Transl Med. (2025) 23:135. doi: 10.1186/s12967-025-06138-6 39885578 PMC11783851

[22] Wahafu W Zhou Q Yang X Yang Y Zhao Y Wang Z . Spatial relationships and interactions of immune cell niches are linked to the pathologic response of muscle-invasive bladder cancer to neoadjuvant therapy. J Transl Med. (2025) 23:375. doi: 10.1186/s12967-025-06358-w 40148849 PMC11948894

[23] Kamat AM Flaig TW Grossman HB Konety B Lamm D O’Donnell MA . Consensus statement on best practice management regarding the use of intravesical immunotherapy with BCG for bladder cancer. Nat Rev Urol. (2015) 12:225–35. doi: 10.1038/nrurol.2015.58 25800393

[24] Mathé G Amiel JL Schwarzenberg L Schneider M Cattan A Schlumberger JR . Active immunotherapy for acute lymphoblastic leukæmia. Lancet. (1969) 293:697–9. doi: 10.1016/S0140-6736(69)92648-8 4182654

[25] Morales A Eidinger D Bruce AW . Intracavitary Bacillus Calmette-guerin in the treatment of superficial bladder tumors. J Urol. (1976) 116:180–2. doi: 10.1016/S0022-5347(17)58737-6 820877

[26] Lamm DL Blumenstein BA Crissman JD Montie JE Gottesman JE Lowe BA . Maintenance Bacillus Calmette-Guerin immunotherapy for recurrent Ta, T1 and carcinoma in situ transitional cell carcinoma of the bladder: a randomized Southwest Oncology Group study. J Urol. (2000) 163:1124–9. doi: 10.1016/S0022-5347(05)67707-5 10737480

[27] Sylvester RJ van der Meijden APM Lamm DL . Intravesical Bacillus Calmette-Guerin reduces the risk of progression in patients with superficial bladder cancer: a meta-analysis of the published results of randomized clinical trials. J Urol. (2002) 168:1964–70. doi: 10.1016/S0022-5347(05)64273-5 12394686

[28] Böhle A Brandau S . Immune mechanisms in Bacillus Calmette-Guerin immunotherapy for superficial bladder cancer. J Urol. (2003) 170:964–9. doi: 10.1097/01.ju.0000073852.24341.4a 12913751

[29] Redelman-Sidi G Glickman MS Bochner BH . The mechanism of action of BCG therapy for bladder cancer—a current perspective. Nat Rev Urol. (2014) 11:153–62. doi: 10.1038/nrurol.2014.15 24492433

[30] Kavoussi LR Brown EJ Ritchey JK Ratliff TL . Fibronectin-mediated Calmette-Guerin bacillus attachment to murine bladder mucosa. Requirement for the expression of an antitumor response. J Clin Invest. (1990) 85:62–7. doi: 10.1172/JCI114434 2404029 PMC296387

[31] Luo Y Yamada H Evanoff DP Chen X . Role of Th1-stimulating cytokines in bacillus Calmette–Guérin (BCG)-induced macrophage cytotoxicity against mouse bladder cancer MBT-2 cells. Clin Exp Immunol. (2006) 146:181–8. doi: 10.1111/j.1365-2249.2006.03191.x 16968412 PMC1809722

[32] Biot C Rentsch CA Gsponer JR Birkhäuser FD Jusforgues-Saklani H Lemaître F . Preexisting BCG-specific T cells improve intravesical immunotherapy for bladder cancer. Sci Transl Med. (2012) 4:137ra72–137ra72. doi: 10.1126/scitranslmed.3003586 22674550

[33] Pichler R Fritz J Zavadil C Schäfer G Culig Z Brunner A . Tumor-infiltrating immune cell subpopulations influence the oncologic outcome after intravesical Bacillus Calmette-Guérin therapy in bladder cancer. Oncotarget. (2016) 7:39916–30. doi: 10.18632/oncotarget.9537 27221038 PMC5129981

[34] Suttmann H Jacobsen M Reiss K Jocham D Böhle A Brandau S . Mechanisms of Bacillus Calmette-Guerin mediated natural killer cell activation. J Urol. (2004) 172:1490–5. doi: 10.1097/01.ju.0000131944.52354.63 15371877

[35] Kleinnijenhuis J Quintin J Preijers F Joosten LAB Ifrim DC Saeed S . Bacille Calmette-Guérin induces NOD2-dependent nonspecific protection from reinfection via epigenetic reprogramming of monocytes. Proc Natl Acad Sci. (2012) 109:17537–42. doi: 10.1073/pnas.1202870109 22988082 PMC3491454

[36] Packiam VT Lamm DL Barocas DA Trainer A Fand B Davis RL . An open label, single-arm, phase II multicenter study of the safety and efficacy of CG0070 oncolytic vector regimen in patients with BCG-unresponsive non–muscle-invasive bladder cancer: interim results. Urologic Oncol Semin Original Investigations. (2018) 36:440–7. doi: 10.1016/j.urolonc.2017.07.005 28755959

[37] Babjuk M Böhle A Burger M Capoun O Cohen D Compérat EM . EAU guidelines on non–muscle-invasive urothelial carcinoma of the bladder: update 2016. Eur Urol. (2017) 71:447–61. doi: 10.1016/j.eururo.2016.05.041 27324428

[38] Chang SS Boorjian SA Chou R Clark PE Daneshmand S Konety BR . Diagnosis and treatment of non-muscle invasive bladder cancer: AUA/SUO guideline. J Urol. (2016) 196:1021–9. doi: 10.1016/j.juro.2016.06.049 27317986

[39] Lobo N Bree KK Hensley PJ Nogueras‐Gonzalez GM Abraham P Navai N . Reduced‐dose bacillus Calmette‐Guérin (BCG) in an era of BCG shortage: real‐world experience from a tertiary cancer centre. BJU Int. (2022) 130:323–30. doi: 10.1111/bju.15661 34847263 PMC11951178

[40] Obara W Eto M Mimata H Kohri K Mitsuhata N Miura I . A phase I/II study of cancer peptide vaccine S-288310 in patients with advanced urothelial carcinoma of the bladder. Ann Oncol. (2017) 28:798–803. doi: 10.1093/annonc/mdw675 27998971

[41] Li R Shah PH Stewart TF Nam JK Bivalacqua TJ Lamm DL . Oncolytic adenoviral therapy plus pembrolizumab in BCG-unresponsive non-muscle-invasive bladder cancer: the phase 2 CORE-001 trial. Nat Med. (2024) 30:2216–23. doi: 10.1038/s41591-024-03025-3 38844794

[42] Saoud R Telfer S Maruf M Singer E Weiss R Jang T . MP16-14 Clinical outcomes of a randomized, prospective, phase II study to determine the efficacy of Bacillus Calmette-Guerin (BCG) given in combination with PANVAC versus BCG given alone in adults with high grade BCG-refractory non-muscle invasive bladder cancer. J Urol. (2021) 206:e302–e302. doi: 10.1097/JU.0000000000002001.14 42348292

[43] Boorjian SA Alemozaffar M Konety BR Shore ND Gomella LG Kamat AM . Intravesical nadofaragene firadenovec gene therapy for BCG-unresponsive non-muscle-invasive bladder cancer: a single-arm, open-label, repeat-dose clinical trial. Lancet Oncol. (2021) 22:107–17. doi: 10.1016/S1470-2045(20)30540-4 33253641 PMC7988888

[44] Sasaki T Ishihara M Kanda H Hori Y Soga N Harada N . PD32-05 Phase I clinical study on the combination therapy of CHP-NY-ESO-1 cancer vaccine and MIS416 for the treatment of patients with NY-ESO-1 expressing refractory urothelial cancer or castration-resistant prostate cancer. J Urol. (2016) 195(4S):e762. doi: 10.1016/j.juro.2016.02.681 38826717

[45] Ott PA Hu-Lieskovan S Chmielowski B Govindan R Naing A Bhardwaj N . A phase Ib trial of personalized neoantigen therapy plus anti-PD-1 in patients with advanced melanoma, non-small cell lung cancer, or bladder cancer. Cell. (2020) 183:347–362.e24. doi: 10.1016/j.cell.2020.08.053 33064988

[46] Di Spirito A Zuccolotto G Tosi A Balkhi S Rosato A Baci D . Evolving frontiers in bladder cancer immunotherapy: integrating BCG, immune checkpoints, viral vectors, nanotechnology, and CAR-based therapies. Front Cell Dev Biol. (2025) 13:1719978. doi: 10.3389/fcell.2025.1719978 41425090 PMC12711830

[47] Balar AV Kamat AM Kulkarni GS Uchio EM Boormans JL Roumiguie M . Pembrolizumab monotherapy for the treatment of high-risk non-muscle-invasive bladder cancer unresponsive to BCG (KEYNOTE-057): an open-label, single-arm, multicentre, phase 2 study. Lancet Oncol. (2021) 22:919–30. doi: 10.1016/S1470-2045(21)00147-9 34051177

[48] Roupret M Bertaut A Pignot G Neuzillet Y Houede N Mathieu R . ALBAN (GETUG-AFU 37): a phase III, randomized, open-label international trial of intravenous atezolizumab and intravesical Bacillus Calmette-Guérin (BCG) versus BCG alone in BCG-naive high-risk, non-muscle-invasive bladder cancer (NMIBC). Ann Oncol. (2026) 37:44–52. doi: 10.1016/j.annonc.2025.09.017 41110692

[49] Grossman HB Natale RB Tangen CM Speights VO Vogelzang NJ Trump DL . Neoadjuvant chemotherapy plus cystectomy compared with cystectomy alone for locally advanced bladder cancer. N Engl J Med. (2003) 349:859–66. doi: 10.1056/NEJMoa022148 12944571

[50] Pfister C Gravis G Fléchon A Chevreau C Mahammedi H Laguerre B . Dose-dense methotrexate, vinblastine, doxorubicin, and cisplatin or gemcitabine and cisplatin as perioperative chemotherapy for patients with nonmetastatic muscle-invasive bladder cancer: results of the GETUG-AFU V05 VESPER trial. J Clin Oncol. (2022) 40:2013–22. doi: 10.1200/JCO.21.02051 35254888

[51] Galsky MD Witjes JA Gschwend JE Milowsky MI Schenker M Valderrama BP . Adjuvant nivolumab in high-risk muscle-invasive urothelial carcinoma: Expanded efficacy from CheckMate 274. J Clin Oncol. (2025) 43:15–21. doi: 10.1200/JCO.24.00340 39393026 PMC11687940

[52] Tateo V Giannatempo P Basile G Proudfoot JA Maiorano BA Cigliola A . Five-year median follow-up update of PURE-01: A phase 2 study of neoadjuvant pembrolizumab followed by radical cystectomy in patients with muscle-invasive bladder cancer (MIBC). J Clin Oncol. (2025) 43:4595. doi: 10.1200/JCO.2025.43.16_suppl.4595 41633898

[53] Meng L Khorasanchi A Jain R . Advancing bladder cancer management: The role of neoadjuvant and adjuvant therapies and biomarkers in muscle invasive bladder cancer. Curr Treat Options Oncol. (2025) 26:929–42. doi: 10.1007/s11864-025-01355-z 41003886

[54] Galsky MD Daneshmand S Izadmehr S Gonzalez-Kozlova E Chan KG Lewis S . Gemcitabine and cisplatin plus nivolumab as organ-sparing treatment for muscle-invasive bladder cancer: A phase 2 trial. Nat Med. (2023) 29:2825–34. doi: 10.1038/s41591-023-02568-1 37783966 PMC10667093

[55] Milowsky MI Galsky MD Witjes JA Gschwend JE Schenker M Perez Valderrama B . Adjuvant nivolumab (NIVO) vs placebo (PBO) for high-risk muscle-invasive urothelial carcinoma (MIUC): Additional efficacy outcomes including overall survival (OS) in patients (pts) with muscle-invasive bladder cancer (MIBC) from CheckMate 274. J Clin Oncol. (2025) 43:658. doi: 10.1200/JCO.2025.43.5_suppl.658

[56] Basile G Bandini M Gibb EA Ross JS Raggi D Marandino L . Neoadjuvant pembrolizumab and radical cystectomy in patients with muscle-invasive urothelial bladder cancer: 3-year median follow-up update of PURE-01 trial. Clin Cancer Res. (2022) 28:5107–14. doi: 10.1158/1078-0432.CCR-22-2158 36190522

[57] Mercinelli C Basile G Proudfoot JA de Jong J Cigliola A Tateo V . Overall survival and biomarker results of NURE-Combo: A phase 2 study of neoadjuvant nivolumab (NIVO) and nab-paclitaxel (ABX) followed by postsurgical adjuvant NIVO in patients (pts) with muscle-invasive bladder cancer (MIBC). J Clin Oncol. (2025) 43:4594. doi: 10.1200/JCO.2025.43.16_suppl.4594 42148471

[58] Raychaudhuri R Khaki AR Redman MW Baker KK Lin A Woo B . Neoadjuvant pembrolizumab (pembro) and accelerated methotrexate, vinblastine, doxorubicin, cisplatin (aMVAC) in non-urothelial (non-UC) histologic subtype muscle invasive bladder cancer (MIBC): A phase 2 trial. J Clin Oncol. (2025) 43:769. doi: 10.1200/JCO.2025.43.5_suppl.769 40764177

[59] Powles T Catto JWF Galsky MD Al-Ahmadie H Meeks JJ Nishiyama H . Perioperative durvalumab with neoadjuvant chemotherapy in operable bladder cancer. N Engl J Med. (2024) 391:1773–86. doi: 10.1056/NEJMoa2408154 39282910

[60] Vulsteke C Adra N Danchaivijitr P Sabadash M Rodriguez-Vida A Zhang Z . Perioperative enfortumab vedotin and pembrolizumab in bladder cancer. N Engl J Med. (2026) 394(13):1257–69. doi: 10.1056/NEJMoa2511674 41707170

[61] Galsky MD Valderrama BP Maruzzo M Font Pous A Ciuleanu T-E Chatzkel JA . Neoadjuvant and adjuvant enfortumab vedotin (EV) plus pembrolizumab (pembro) for participants with muscle-invasive bladder cancer (MIBC) who are eligible for cisplatin: Randomized, open-label, phase 3 KEYNOTE-B15 study. J Clin Oncol. (2026) 44:LBA630–LBA630. doi: 10.1200/JCO.2026.44.7_suppl.LBA630 42148471

[62] Maiorano BA Tateo V Cigliola A Mercinelli C Briganti A Montorsi F . Perioperative enfortumab vedotin plus pembrolizumab versus neoadjuvant pembrolizumab in cisplatin-ineligible patients with muscle-invasive bladder cancer (MIBC): An IPD-based comparative analysis of KEYNOTE-905/EV-303 and PURE-01. J Clin Oncol. (2026) 44:779. doi: 10.1200/JCO.2026.44.7_suppl.779

[63] James ND Hussain SA Hall E Jenkins P Tremlett J Rawlings C . Radiotherapy with or without chemotherapy in muscle-invasive bladder cancer. N Engl J Med. (2012) 366:1477–88. doi: 10.1056/NEJMoa1106106 22512481

[64] Powles T Kann AG Castellano D Gross-Goupil M Nishiyama H Bracarda S . ctDNA-guided adjuvant atezolizumab in muscle-invasive bladder cancer. N Engl J Med. (2025) 393:2395–408. doi: 10.1056/NEJMoa2511885 41124204

[65] Ghatalia P Ross EA Zibelman MR Anari F Abbosh P Tester WJ . A phase 2 trial of risk enabled therapy after neoadjuvant chemo-immunotherapy for muscle-invasive bladder cancer (RETAIN-2). J Clin Oncol. (2025) 43:815. doi: 10.1200/JCO.2025.43.5_suppl.815 PMC11908952 39680823

[66] Geynisman DM Abbosh PH Ross E Zibelman MR Ghatalia P Anari F . Phase II trial of risk-enabled therapy after neoadjuvant chemotherapy for muscle-invasive bladder cancer (RETAIN 1). J Clin Oncol. (2025) 43:1113–22. doi: 10.1200/JCO-24-01214 39680823 PMC11908952

[67] Sternberg CN Yagoda A Scher HI Watson RC Geller N Herr HW . Methotrexate, vinblastine, doxorubicin, and cisplatin for advanced transitional cell carcinoma of the urothelium. Efficacy and patterns of response and relapse. Cancer. (1989) 64:2448–58. doi: 10.1002/1097-0142(19891215)64:12<2448::aid-cncr2820641209>3.0.co;2-7 2819654

[68] Sternberg CN de Mulder PHM Schornagel JH Théodore C Fossa SD van Oosterom AT . Randomized phase III trial of high–dose-intensity methotrexate, vinblastine, doxorubicin, and cisplatin (MVAC) chemotherapy and recombinant human granulocyte colony-stimulating factor versus classic MVAC in advanced urothelial tract tumors: European Organization for Research and Treatment of Cancer Protocol No. 30924. J Clin Oncol. (2001) 19:2638–46. doi: 10.1200/JCO.2001.19.10.2638 11352955

[69] Powles T Valderrama BP Gupta S Bedke J Kikuchi E Hoffman-Censits J . Enfortumab vedotin and pembrolizumab in untreated advanced urothelial cancer. N Engl J Med. (2024) 390:875–88. doi: 10.1056/NEJMoa2312117 38446675

[70] van der Heijden MS Sonpavde G Powles T Necchi A Burotto M Schenker M . Nivolumab plus gemcitabine–cisplatin in advanced urothelial carcinoma. N Engl J Med. (2023) 389:1778–89. doi: 10.1056/NEJMoa2309863 37870949 PMC12314471

[71] Vuky J Balar AV Castellano D O’Donnell PH Grivas P Bellmunt J . Long-term outcomes in KEYNOTE-052: Phase II study investigating first-line pembrolizumab in cisplatin-ineligible patients with locally advanced or metastatic urothelial cancer. J Clin Oncol. (2020) 38:2658–66. doi: 10.1200/JCO.19.01213 32552471

[72] Powles T Park SH Voog E Caserta C Valderrama BP Gurney H . Avelumab maintenance therapy for advanced or metastatic urothelial carcinoma. N Engl J Med. (2020) 383:1218–30. doi: 10.1056/NEJMoa2002788 32945632

[73] Bellmunt J de Wit R Vaughn DJ Fradet Y Lee J-L Fong L . Pembrolizumab as second-line therapy for advanced urothelial carcinoma. N Engl J Med. (2017) 376:1015–26. doi: 10.1056/NEJMoa1613683 28212060 PMC5635424

[74] Sharma P Retz M Siefker-Radtke A Baron A Necchi A Bedke J . Nivolumab in metastatic urothelial carcinoma after platinum therapy (CheckMate 275): a multicentre, single-arm, phase 2 trial. Lancet Oncol. (2017) 18:312–22. doi: 10.1016/S1470-2045(17)30065-7 28131785

[75] Patel MR Ellerton J Infante JR Agrawal M Gordon M Aljumaily R . Avelumab in metastatic urothelial carcinoma after platinum failure (JAVELIN Solid Tumor): pooled results from two expansion cohorts of an open-label, phase 1 trial. Lancet Oncol. (2018) 19:51–64. doi: 10.1016/S1470-2045(17)30900-2 29217288 PMC7984727

[76] Powles T Durán I van der Heijden MS Loriot Y Vogelzang NJ De Giorgi U . Atezolizumab versus chemotherapy in patients with platinum-treated locally advanced or metastatic urothelial carcinoma (IMvigor211): a multicentre, open-label, phase 3 randomised controlled trial. Lancet. (2018) 391:748–57. doi: 10.1016/S0140-6736(17)33297-X 29268948

[77] Powles T van der Heijden MS Castellano D Galsky MD Loriot Y Petrylak DP . Durvalumab alone and durvalumab plus tremelimumab versus chemotherapy in previously untreated patients with unresectable, locally advanced or metastatic urothelial carcinoma (DANUBE): a randomised, open-label, multicentre, phase 3 trial. Lancet Oncol. (2020) 21:1574–88. doi: 10.1016/S1470-2045(20)30541-6 32971005

[78] Strati A Adamopoulos C Kotsantis I Psyrri A Lianidou E Papavassiliou AG . Targeting the PD-1/PD-L1 signaling pathway for cancer therapy: Focus on biomarkers. Int J Mol Sci. (2025) 26:1235. doi: 10.3390/ijms26031235 39941003 PMC11818137

[79] Ritterhouse LL . Tumor mutational burden. Cancer Cytopathol. (2019) 127:735–6. doi: 10.1002/cncy.22174 31433548

[80] Boll LM Perera-Bel J Rodriguez-Vida A Arpí O Rovira A Juanpere N . The impact of mutational clonality in predicting the response to immune checkpoint inhibitors in advanced urothelial cancer. Sci Rep. (2023) 13:15287. doi: 10.1038/s41598-023-42495-2 37714872 PMC10504302

[81] Powles T Van Der Heijden MS Wang Y Catto JWF Meeks JJ Al-Ahmadie H . Circulating tumor DNA (ctDNA) in patients with muscle-invasive bladder cancer (MIBC) who received perioperative durvalumab (D) in NIAGARA. J Clin Oncol. (2025) 43:4503. doi: 10.1200/JCO.2025.43.16_suppl.4503 42148471

[82] Ghatalia P Ross EA Zibelman MR Anari F Abbosh P Herberts C . Circulating tumor DNA (ctDNA) to guide response-adapted bladder preservation in muscle invasive bladder cancer (MIBC): Integrated analysis of the RETAIN trials. J Clin Oncol. (2026) 44:LBA632–LBA632. doi: 10.1200/JCO.2026.44.7_suppl.LBA632 42148471

[83] Wang X He J Ding G Tang Y Wang Q . Artificial intelligence-enabled multi-omics for predicting immune checkpoint inhibitor response and resistance. J Multidiscip Healthc. (2026) 19:572089. doi: 10.2147/JMDH.S572089 41777263 PMC12951862

[84] Wang X Xiong D Cui S Duan B Ding G Huang Y . Artificial intelligence-enabled multi-omics biomarkers for immune checkpoint blockade: Mechanisms, predictive modeling, and clinical translation. Front Immunol. (2026) 17:1732079. doi: 10.3389/fimmu.2026.1732079 41808840 PMC12967935

[85] Alberca-Del Arco F Santos-Perez de la Blanca R Matas-Rico EM Herrera-Imbroda B Guerrero-Ramos F . Emerging molecular and computational biomarkers in urothelial carcinoma: Innovations in diagnosis, prognosis, and therapeutic response prediction. J Pers Med. (2026) 16:25. doi: 10.3390/jpm16010025 41590518 PMC12843400

[86] Seo HK Lee HW Sa J Cho HJ Koo H Jung EH . Comprehensive multi-layered omics analysis of the UBC landscape for precision medicine and its clinical implications: From deciphering distinct molecular heterogeneity to guiding treatment decision-making and developing tailored therapeutic strategies. J Clin Oncol. (2026) 44:840. doi: 10.1200/JCO.2026.44.7_suppl.840 41701950

[87] Zhang J Wang Y Yan Q Wang H Ran Q Zhu H . SWI/SNF complex alterations predict immunotherapy response in bladder cancer. Front Immunol. (2025) 16:1708324. doi: 10.3389/fimmu.2025.1708324 41438758 PMC12719508

[88] Rafii S Mukherji D Komaranchath AS Khalil C Iqbal F Abdelwahab SI . Advancing CAR T-cell therapy in solid tumors: Current landscape and future directions. Cancers (Basel). (2025) 17:2898. doi: 10.3390/cancers17172898 40940995 PMC12428560

[89] Martinez M Moon EK . CAR T cells for solid tumors: New strategies for finding, infiltrating, and surviving in the tumor microenvironment. Front Immunol. (2019) 10. doi: 10.3389/fimmu.2019.00128 30804938 PMC6370640

[90] Luo N Chen C Zhou W Hao J He S Liu Y . Natural killer cell-mediated antitumor immunity: Molecular mechanisms and clinical applications. MedComm (Beijing). (2025) 6:e70387. doi: 10.1002/mco2.70387 40959206 PMC12433904

[91] Fabian KP Padget MR Donahue RN Solocinski K Robbins Y Allen CT . PD-L1 targeting high-affinity NK (t-haNK) cells induce direct antitumor effects and target suppressive MDSC populations. J Immunother Cancer. (2020) 8:e000450. doi: 10.1136/jitc-2019-000450 32439799 PMC7247398

[92] Zhang S Zhao J Bai X Handley M Shan F . Biological effects of IL-15 on immune cells and its potential for the treatment of cancer. Int Immunopharmacol. (2021) 91:107318. doi: 10.1016/j.intimp.2020.107318 33383444

[93] Zheng X Chen Y Dong G Zhang Y Zhang M An B . Abstract 3427: HY07121, a new generation of immune oncology therapeutics overcoming tumor immune resistance. Cancer Res. (2025) 85:3427. doi: 10.1158/1538-7445.AM2025-3427 36230740

[94] Tang K Wu Y-H Song Y Yu B . Indoleamine 2,3-dioxygenase 1 (IDO1) inhibitors in clinical trials for cancer immunotherapy. J Hematol Oncol. (2021) 14:68. doi: 10.1186/s13045-021-01080-8 33883013 PMC8061021

[95] Shafren D Lee C Peters J Tay S Evans C Quah M . Abstract 859: Novel oncolytic RNA viruses, IVX037 and IVX055, demonstrate potent antitumor activity. Cancer Res. (2025) 85:859. doi: 10.1158/1538-7445.AM2025-859 39804967

[96] Zhang C Liu S Zhang J Lu J Chen Z Pan B . A multifunctional Fe-EGCG@RSL3 nanomedicine synergizes ferroptosis induction and tumor microenvironment remodeling for enhanced bladder cancer immunotherapy. Research. (2025) 8:0735. doi: 10.34133/research.0735 40530387 PMC12173478

[97] Motallebzadeh Khanmiri J Khani-Eshratabadi M Seyedmoharrami F Khazaee-Nasirabadi MH Dehdashti M Seddighi N . Innovative combinatory approaches with dendritic cell-based vaccines: Bridging preclinical insights and clinical challenges. Clin Exp Med. (2026) 26:154. doi: 10.1007/s10238-026-02056-z 41689608 PMC12909375

[98] Xiao J Liu J Zhang C Liu Z Nie Z Yi Z . Overcoming immunotherapy resistance in bladder cancer with a novel antibody-drug conjugate RC48. J Immunother Cancer. (2025) 13(8):e011881. doi: 10.1136/jitc-2025-011881 40789740 PMC12352158

[99] Chen J Fu Y Zhang Z Zhao J Zuo J Ye X . Nanomaterial-enhanced immunotherapy: Advancing T-cell-based treatments for bladder cancer. Int J Nanomedicine. (2025) 20:15235–75. doi: 10.2147/IJN.S557690 41438430 PMC12721131

[100] Huang X Gu R Li Z Wang F . Igniting cold tumors: Multi-omics-driven strategies to overcome immune evasion and restore immune surveillance. Oncol Res. (2025) 33:2857–902. doi: 10.32604/or.2025.066805 41050070 PMC12494119

[101] Jain P Naqvi SAA Tripathi N Oberoi JK Humayun MA Zakharia Y . Real-world outcomes of enfortumab vedotin and pembrolizumab in advanced urothelial carcinoma: A multicenter retrospective analysis. Clin Genitourin Cancer. (2025) 23:102453. doi: 10.1016/j.clgc.2025.102453 41177680

